# Is the bacterial leaf nodule symbiosis obligate for *Psychotria umbellata*? The development of a *Burkholderia*-free host plant

**DOI:** 10.1371/journal.pone.0219863

**Published:** 2019-07-16

**Authors:** Arne Sinnesael, Olivier Leroux, Steven B. Janssens, Erik Smets, Bart Panis, Brecht Verstraete

**Affiliations:** 1 Plant Conservation and Population Biology, Department of Biology, KU Leuven, Leuven, Belgium; 2 Meise Botanic Garden, Meise, Belgium; 3 Department of Biology, Ghent University, Ghent, Belgium; 4 Naturalis Biodiversity Center, Leiden, the Netherlands; 5 Institute of Biology Leiden, Leiden University, Leiden, the Netherlands; 6 Bioversity International, Leuven, Belgium; 7 Natural History Museum, University of Oslo, Oslo, Norway; Centre National de la Recherche Scientifique, FRANCE

## Abstract

**Background & aims:**

The bacterial leaf nodule symbiosis is an interaction where bacteria are housed in specialised structures in the leaves of their plant host. In the Rubiaceae plant family, host plants interact with *Burkholderia* bacteria. This interaction might play a role in the host plant defence system. It is unique due to its high specificity; the vertical transmission of the endophyte to the next generation of the host plant; and its supposedly obligatory character. Although previous attempts have been made to investigate this obligatory character by developing *Burkholderia*-free plants, none have succeeded and nodulating plants were still produced. In order to investigate the obligatory character of this endosymbiosis, our aims were to develop *Burkholderia-*free *Psychotria umbellata* plants and to investigate the effect of the absence of the endophytes on the host in a controlled environment.

**Methods:**

The *Burkholderia-*free plants were obtained via embryo culture, a plant cultivation technique. In order to analyse the endophyte-free status, we screened the plants morphologically, microscopically and molecularly over a period of three years. To characterise the phenotype and growth of the *in vitro* aposymbiotic plants, we compared the growth of the *Burkholderia*-free plants to the nodulating plants under the same *in vitro* conditions.

**Key results:**

All the developed plants were *Burkholderia*-free and survived in a sterile *in vitro* environment. The growth analysis showed that plants without endophytes had a slower development.

**Conclusions:**

Embryo culture is a cultivation technique with a high success rate for the development of *Burkholderia*-free plants of *P*. *umbellata*. The increased growth rate *in vitro* when the specific endophyte is present cannot be explained by possible benefits put forward in previous studies. This might indicate that the benefits of the endosymbiosis are not yet completely understood.

## Introduction

A plant is not a sterile entity, but interacts with many microorganisms, including fungi, protozoans and bacteria [1–4]. Many of these interactions are not pathogenic, but ameliorate the fitness of the host by facilitating nutrient uptake (e.g., mycorrhizal fungi, rhizobia), by increasing resistance to abiotic or biotic stress (alkaloid producing fungi) or by stimulating growth or germination [1,5–10]. Despite the importance of these interactions, the potential benefits for some specific hosts and/or endophytes remain unclear [4,11–13]. New molecular tools may help towards identifying possible benefits for the host; for example, genomic tools identified possible functional benefits of the microbiome for *Arabidopsis thaliana* [14,15], and transcriptomics increased the knowledge on the communication between the plant-growth promoting *Burkholderia* Q208 and sugarcane [16]. Despite the progress made, identifying the benefits of the interaction is still challenging. While most of the functional genes were discovered in axenic cultures of the endophytes [4,15,17], some endophytes cannot be cultivated as pure cultures [1,4,11,12]. Furthermore, the natural environment is much more complex than one-on-one interactions. Hosts interact with multiple endophytes, and endophytes can have more than one benefit or even differ in benefits depending on the environment. These diverse possibilities make it challenging to disentangle the effect of the endophytes on the phenotype of the host [1,4,7,11,12,17,18].

Bacterial leaf nodule symbiosis is an interaction between bacteria and host plant species, characterised by the occurrence of the endophytes in structured cavities in the leaves, visible as nodules [19–22]. It has been identified in three flowering plant families, i.e. Dioscoreaceae, Primulaceae and Rubiaceae, and it has been suggested that they also occur in Styracaceae [20,21,23,24]. This intimate interaction is unique in angiosperms due to its high specificity and the presence of vertical transmission [19–21,24,25]. In addition to its high specificity and its (mainly) vertical transmission, leaf nodulation is suggested to be obligate for both partners [19–21]. In Rubiaceae, culture-independent methods were necessary to identify the nodulated endophytes as *Burkholderia* [21,26,27], because endosymbionts that occur with this genus are commonly unculturable outside the plant hosts [19,20,28]. Naturally nodule-free host plants can occur in small numbers when *Psychotria punctata* (a synonym of *P*. *kirkii* [29]) are cultivated from seed [20,30]. In contrast to the nodulating plants, these plants cease their growth and development after the second or third leaf pair [20,30]. To further investigate the obligatory nature of the interaction, several studies have attempted to produce a nodule-free plant by exposing seedlings to hot and dry conditions [20,31,32]. These seedlings displayed a ‘crippled’ phenotype with distorted leaves and a stunted appearance, and lacked the characteristic nodules. However, after placing the plants under optimal growing conditions, leaf nodules appeared on the new lateral branches [31], suggesting that they were not truly *Burkholderia-*free. This indicates that the observation of nodule-free leaves is not enough to confirm the symbiont-free status of the host plant. For this reason, morphological observations should ideally be complemented with microscopic and molecular analyses in order to confirm the *Burkholderia-*free status of the plants.

Despite the uniqueness of the bacterial leaf nodule symbiosis, the benefits of the interaction are still not clear. Although morphological observations of nodule-free seedlings seemed to suggest that the endophyte is beneficial for the growth and development of the host [20,31], later studies using molecular tools pointed towards a role in protection against herbivory [28,33,34]. If the main function of the endophytes is indeed solely providing protection against herbivory, the endophytic presence should not be obligate for the host plant when cultivated under controlled conditions (*in vitro* and greenhouse conditions). Furthermore, the aposymbiotic phenotype should be similar to the natural nodulating phenotype under these conditions. In this study, our aim was to develop *Burkholderia-*free *Psychotria umbellata* plants using the embryo culture. These aposymbiotic plants were further used to assess the obligatory nature of the nodule-forming endosymbiont of *Psychotria umbellata*.

## Materials and methods

### Embryo culture of *Psychotria umbellata* (EC plants) and the natural *Burkholderia-*free *P*. *nervosa*

The technique of embryo culture was applied to produce *Burkholderia*-free *Psychotria umbellata* by isolating plant embryos from seeds and growing them *in vitro* on a nutrient-rich medium. It is commonly used to reduce the dormancy period, or to avoid germination difficulties caused by disease, endophytic dependence or embryogenic failure [35–38]. Embryo culture (EC) was chosen because: (1) the endophytes are present in the seeds, but they have not infected the embryos [25]; and (2) the number of endophytes in the seeds is low [25,30], which reduces the risk of accidentally transferring endophytes.

**Fig 1:**
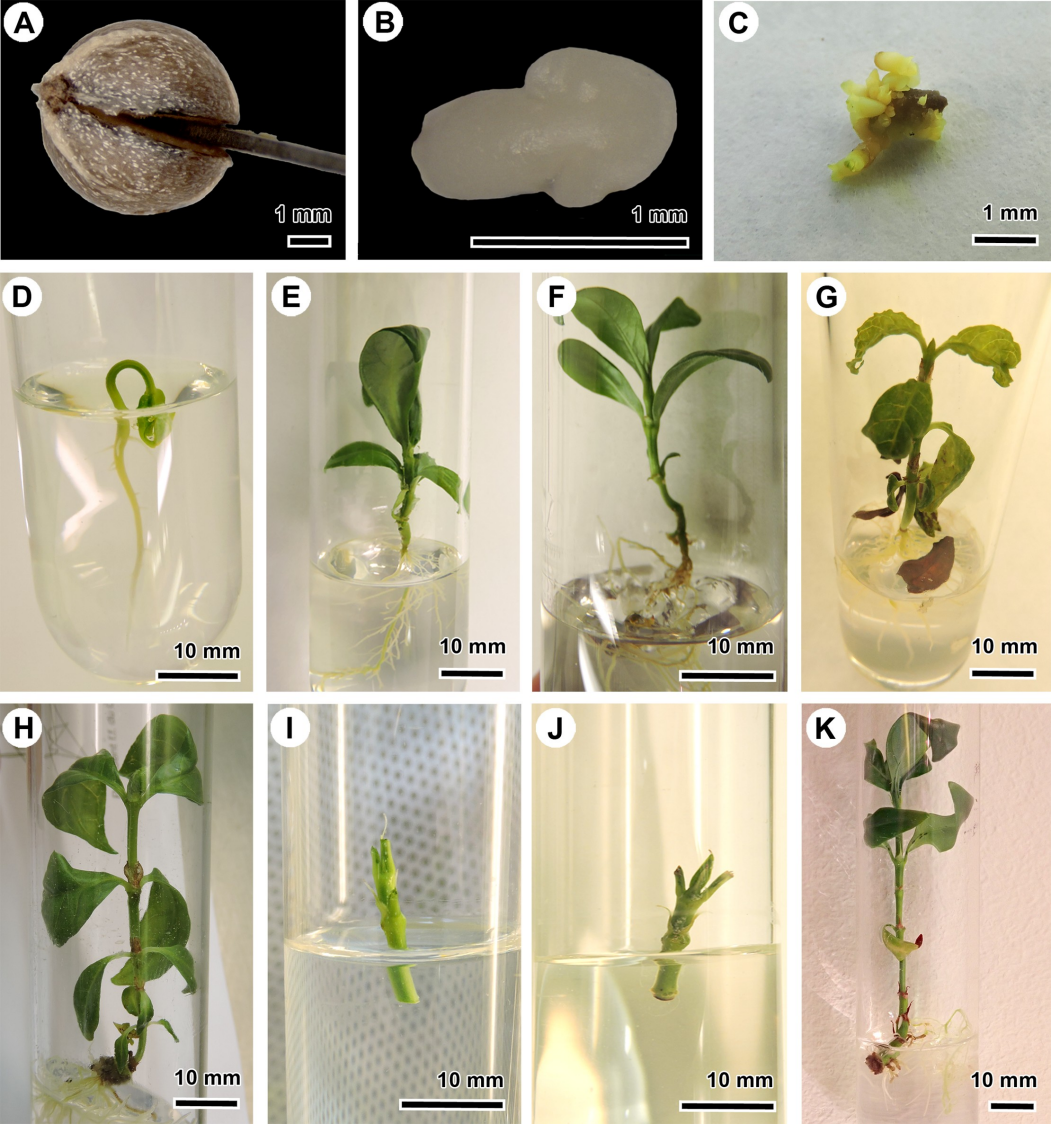
*In vitro* cultivation of *Psychotria umbellata* and natural endophyte-free *Psychotria nervosa* using embryo culture and shoot culture. (A-H) Illustration of the technique embryo culture. (A) Example of an incision in the pyrene of *P*. *umbellata* along the longitudinal axis away from the micropyle to expose the embryo. (B) The isolated embryo of *P*. *umbellata* before sterile transfer to nutrient-rich plant medium. (C) Adventive embryony on an embryo of *P*. *umbellata*. (D) Two-week-old *P*. *umbellata* seedling obtained via embryo culture. (E) Three-month-old *P*. *umbellata* plantlet obtained via embryo culture. (F) Shoot from a 2.5-year-old *P*. *umbellata* plant obtained via embryo culture. (G) Three-month-old *P*. *nervosa* plantlet obtained via embryo culture. (H) Shoot from a 2.25-year-old *P*. *nervosa* culture obtained via embryo culture. (I-K) Illustration of the technique of shoot culture. (I) Example of a sterilized cutting transferred to *in vitro* culture medium of *P*. *umbellata*. (J) Two-week-old *P*. *umbellata* plantlet obtained via shoot culture. (K) Eight-month-old *P*. *umbellata* plantlet with nodules obtained via shoot culture.

We collected 300 fresh stone fruits (drupes) of *Psychotria umbellata* from the living collection at Meise Botanic Garden. All drupes were collected from one shrub (accession number 19620512) to minimize genetic differences. In addition, we collected 30 fruits of the natural *Burkholderia-*free *P*. *nervosa* (20070329–59) to use as a control to screen for putative effects of the *in vitro* methodology. We removed the fleshy mesocarp and stored the pyrenes (seeds encapsulated by a hardened endocarp) at 6°C and 10% relative air humidity (Fig 1A).

We sterilised the surface of the pyrenes under a laminar flow by subsequently treating them with 96% (v/v) ethanol for three minutes; 1.6% (w/v) sodium hypochlorite with 0.1% (v/v) Tween20 for 20 minutes; and by rinsing them with sterile water. We excised the embryos by making an incision along the longitudinal axis of the pyrene away from the micropyle (Fig 1A). Subsequently, the pyrene was ruptured along the same axis, exposing the embryo (Fig 1B). In total, 240 embryos of *P*. *umbellata* and 24 of *P*. *nervosa* (control) were isolated and transferred to nutrient-rich sterile ½ Murashige and Skoog (MS) medium in sealed test tubes [39]. We grew the embryos in dark conditions at 27°C during the first month to avoid rapid germination and photo-oxidation [40], and subsequently transferred them to day/night conditions (16 h light and 8 h dark period) under white fluorescent lamps at 25±2°C [41]. Each month, we transferred the EC plantlets to fresh medium to avoid nutrient depletion and checked for the presence of leaf nodules. If abundant axillary shoots were present, some of them were used as cuttings and transferred to new sealed tubes to increase the number of plantlets (i.e., shoot multiplication). We micropropagated the plantlets for three years to ensure that these plantlets remained in their putative endophyte-free status.

### *In vitro* shoot culture of nodulating *Psychotria umbellata* (SC plants)

We used a second *in vitro* technique to develop nodulating plants under the same *in vitro* conditions as the EC plantlets. To this end, we collected twigs of nodulating *Psychotria umbellata* from one shrub at Meise Botanic Garden and divided the twigs in plant cuttings containing two nodes each. We surface sterilised 40 plant cuttings with 96% (v/v) ethanol (three minutes), 1.6% (w/v) sodium hypochlorite (10 minutes) and subsequently rinsed them three times with sterile water. To improve the uptake of nutrients, the outer ends of the cuttings were trimmed to remove possible damage caused by the sterilising agents. We subsequently transferred the cuttings to nutrient-rich ½ MS plant cultivation medium under a laminar flow (Fig 1I). Each month, these SC plantlets were transferred to new plant cultivation medium. We preferred this cutting technique over *in vitro* seed germination to avoid possible delays due to seed dormancy. We used these nodulating *in vitro* plantlets as positive control to visualise possible phenotypic differences between the putative *Burkholderia*-free plant and the natural nodulating phenotype under *in vitro* conditions. If abundant axillary shoots were present, shoot multiplication was also used to increase the number of plantlets.

### Morphological analysis

Once a month, we screened the EC and SC plantlets for the presence of nodules and monitored them over a period of three years. To assess the presence or absence of leaf nodules, we investigated the plantlets microscopically after three years. We collected three leaves of equal size from each of the two different plant types (EC, SC). To assure that the cultivation technique and the small leaf size did not influence the growth and development of the nodules, three leaves, equal in size of those of the *in vitro* plantlets, were added from an adult plant at Meise Botanic Garden. All leaves were fixed in 4% (v/v) formaldehyde in PEM buffer (100 mM 1,4-piperazinediethanesulfonic acid, 10 mM MgSO_4_, and 10 mM ethylene glycol tetra-acetic acid, pH 6.9), rinsed in water and subsequently analysed with a Nikon SMZ800 stereo microscope (equipped with a Nikon DS-Ri1 camera). Subsequently, we dissected a small part of the leaf blades along the midvein containing nodules and embedded it in 8% agarose. In case no obvious nodules were visible, we dissected a small part of the leaf blades along the midvein close to the petiole. This region is where nodules would occur under natural circumstances. After hardening, we glued the agarose-embedded samples to the vibratome stage with superglue (Roticoll, Carl Roth, Karlsruhe, Germany) and made series of 30 μm sections with a vibrating microtome (HM650V, Thermo Fisher Scientific, Waltham, MA, USA). Afterwards, sections were stained for three minutes with 0.5% (w/v) astra blue, 0.5% (w/v) chrysoidine and 0.5% (w/v) acridine red. These were then rinsed with water, dehydrated with isopropyl alcohol and subsequently mounted in Euparal. We observed the sections with a Nikon Eclipse Ni-U bright field microscope equipped with a Nikon DS-Filc camera.

### Molecular analysis

We preserved a leaf of each EC and SC plantlet in silica gel in each of year 1, 2 and 3. Dried leaves were pulverised with a tissue homogeniser and total genomic DNA was extracted using a modified CTAB protocol [42]. We subsequently tested whether endophytic DNA could be detected using the specific bacterial primers 16S rDNA [43], *recA* and *gyrB* [44]. To reduce the possibility of false negatives, we added two positive controls (a leaf sample of a nodulating *P*. *umbellata* and a culture of the soil bacteria *Burkholderia caledonica*) and one negative control in each PCR run. In addition, we tested for the presence of plant DNA with plant primers for *trnL-F* [45].

When bacterial DNA was detected in samples of the EC plantlets, the PCR products were purified and bidirectionally sequenced by Macrogen Facilities (Macrogen Europe, Amsterdam, the Netherlands). Subsequently, the sequences were assembled and compared with sequences on GenBank with the online BLAST tool.

### Phenotypic analyses

#### *In vitro* phenotype

To assess the effect of the endophyte on the growth of the *Psychotria* host, we compared the embryo-cultured (putative *Burkholderia*-free) plantlets to the *in vitro* cultivated shoots (containing *Burkholderia*). To avoid the influence of the different cultivation techniques (embryo culture vs. shoot culture), we applied the same cultivation technique on both EC and SC plantlets. Shoots (shoot apical meristem and one node) of 24 SC plantlets and of 40 EC plantlets were obtained, and transferred to fresh nutrient-rich medium (Fig 1I) (shoot multiplication). During the next four months, we monitored the growth every two weeks by measuring the length starting from the first node and counting the developing nodes. The differences in length and the number of developing nodes were statistically assessed using a non-parametric Wilcoxon rank test in R [46].

#### Phenotype under greenhouse conditions

Due to the presence of a high sugar concentration and the poor light conditions, the *in vitro* phenotype can differ from the natural phenotype [47]. In order to evaluate for possible *in vitro* effects, we transferred 11 EC and 2 SC plantlets of *P*. *umbellata*, and 3 EC plantlets of *P*. *nervosa* to greenhouse conditions (16 hours light at 26°C and 80% humidity, 8 hours dark at 16°C and 70% humidity). All plantlets were selected based on their size (>40 mm) and the presence of roots and a minimum of four leaves. The plantlets were planted in pots containing soil mixed with 15% Rhine sand and covered with a plastic cover to increase the air humidity. To acclimatize these plants to less humid conditions, the plastic cover was gradually removed after four weeks. The plants were monitored every month to analyse their growth by counting the extra nodes or axillary shoots and their survival.

## Results

### Micropropagation

Two months after embryo rescue, 58% of the embryos of *Psychotria umbellata* developed into seedlings (Fig 1D). Additionally, adventive embryony was observed in 27% of the developing embryos of *P*. *umbellata*. These were transferred to new sealed tubes and thereby increased the number of obtained seedlings (Fig 1C). Neither bacterial nor fungal contamination was observed in any of the *in vitro* tubes. After one year, only 35% of the seedlings survived, and developed new leaves and a root system (Fig 1C–1F). Most of the surviving plantlets originated from adventive embryogenesis (i.e., new embryo clones developed from the somatic cells of an embryo; 452 clones derived from 20 original embryos) or via shoot multiplication (572 clones derived from 30 original embryos). After three years, 12% of the original embryos survived of which three quarters were multiplied via adventive embryogenesis (generating 123 clones) and one quarter were multiplied via shoot multiplication (18 clones).

For the natural *Burkholderia-*free *P*. *nervosa*, 100% of the embryos developed to seedlings. In contrast to *P*. *umbellata*, adventive embryony was not observed. After one year, 70% of the original embryos survived and, due to their fast growth, generated 48 clones via shoot multiplication (Fig 1G). After three years, 30% of the original embryos survived, generating 23 clones (Fig 1H).

Two months after the transfer of the nodulating plant cuttings of *P*. *umbellata*, 26 out of 40 of the SC plantlets survived (Fig 1I–1K). In contrast to the EC plantlets, the leaves of 24 of the surviving SC plantlets had nodules along the midvein, visible on the abaxial leaf surface (Fig 1K). The other two of the surviving SC plantlets were nodule-free.

### Morphological analysis

**Fig 2:**
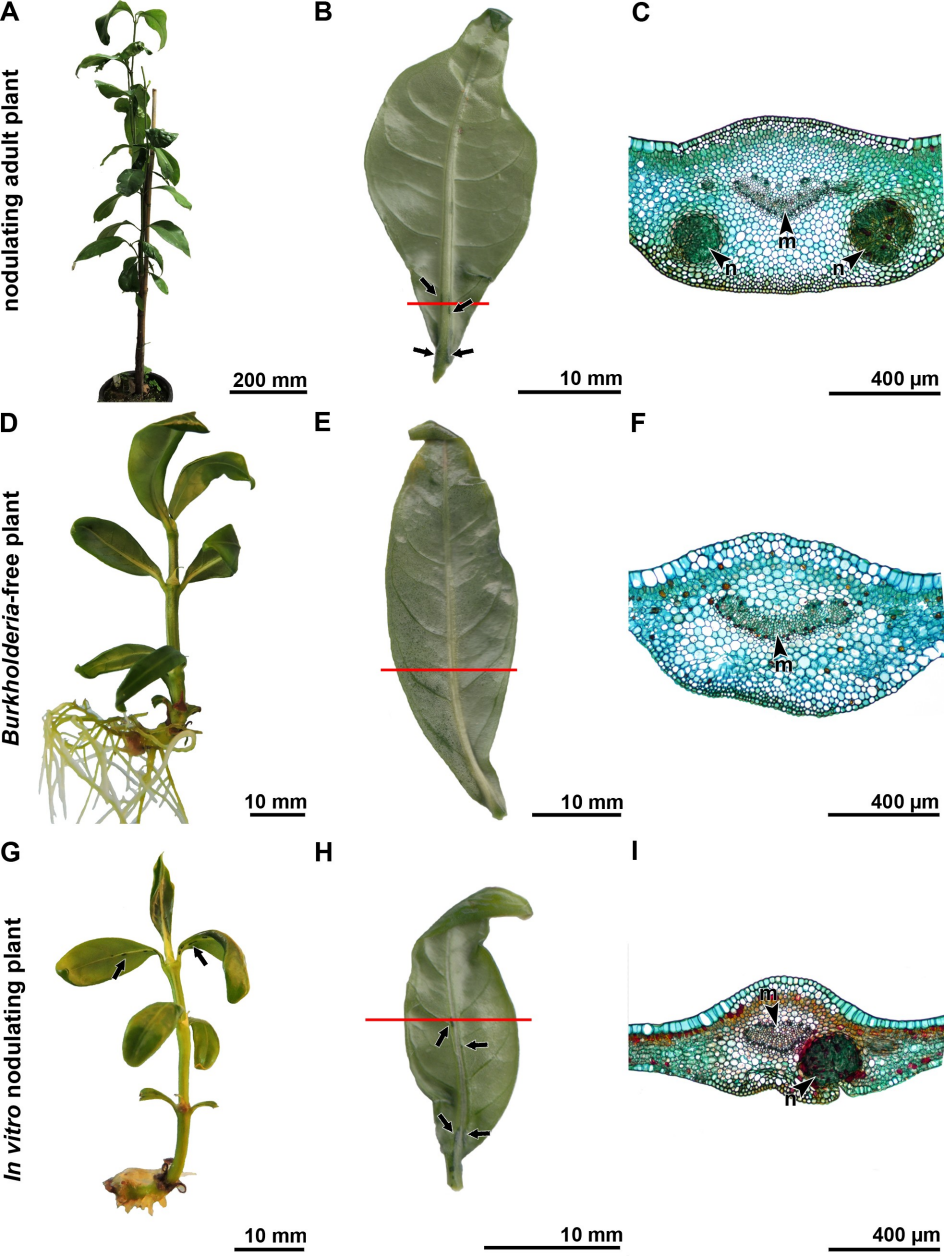
Morphological and microscopic analysis of an adult plant, *in vitro* nodulating SC and *in vitro* nodule-free EC plantlets of *Psychotria umbellata*. (A-C) Woody adult plant in Meise Botanic Garden. (A) Overview of the adult plant. (B) Abaxial lamina surface of a young leaf (sampled of A) showing the presence of nodules (arrows) and the site of the section (red line). (C) Stained vibratome section of the leaf (B) showing the presence of two nodules in the spongy mesophyll close to the midvein. (D-F) *In vitro* cultivated *Burkholderia-*free plantlet obtained via embryo culture. (D) Overview of an *in vitro* EC plant. (E) Abaxial lamina surface (obtained of an *in vitro Burkholderia*-free plantlet) without nodules, showing the site of section (red line). (F) Stained vibratome section of the leaf (E) where no structured cavities or nodules are detected. (G-I) *In vitro* cultivated plant cutting. (G) *In vitro* SC plant showing the presence of nodules (arrows). (H) Abaxial lamina surface (obtained of an *in vitro* nodulating plantlet) showing the presence of nodules (arrows) and the site of the section (red line). (I) Stained vibratome section of the leaf (H) showing the presence of a nodule in the spongy mesophyll close to the midvein. n, nodule; m, midvein.

The nodules of *P*. *umbellata* are dark elongated structures positioned along the midvein with the highest density closest to the petiole (Fig 2A and 2B). The nodules are embedded in the spongy parenchyma of the mesophyll, and the cavity, containing the endophytes, is enclosed by two or three cell layers of compressed mesophyll (Fig 2C).

Every month, we investigated each EC plantlet of *P*. *umbellata* macroscopically for the presence of nodules on the abaxial side of the leaves, yet these specialised structures were never observed (Fig 2D and 2E). We analysed the nodule-free leaves microscopically as well to confirm the absence of smaller or less-developed nodules. From these plantlets (two obtained by adventive embryony and one via subculture), we selected a leaf region close to the petiole where normally the highest number of nodules can be observed in infected plants, but no structured cavities or nodules were detected in the leaf lamina (Fig 2F).

In contrast to the EC plantlets of *P*. *umbellata*, we observed elongated nodules in the leaves of the SC plantlets, close to the petiole and along the midvein in the spongy parenchyma, as well as in the leaves of the woody adult plants growing at Meise Botanic Garden (Fig 2G–2I).

Besides the bacterial nodules, dark protruding structures were observed on leaves of *P*. *umbellata*. These structures could be differentiated from the bacterial nodules by their less elongated shape, and they were not located close to the midvein but instead scattered over the leaf lamina. These spots were also observed on the leaves of nodule-free *P*. *umbellata* plantlets (S1B Fig). Transverse sections through these structures showed that these were protrusions of plant tissue (S1C Fig). On the nodulating leaves of adult plants, these protruding dark spots were also observed (S1D Fig). One of the most prominent protruding spots was analysed microscopically. Transverse sections through these structures indicated that these were protrusions of leaf tissue due to the local development of periderm tissue. Taken together, none of the aforementioned structures are bacterial nodules (S1E and S1F Fig).

### Molecular analysis

During the three-year monitoring, we extracted DNA from whole leaves of 88 EC plants. This procedure was repeated three times on new leaves of the same EC plants. Endophytic DNA was never detected with the specific 16S rDNA, *recA* and *gyrB* primers in these EC plantlets (Table 1). Furthermore, we collected and analysed shoot tips of six of these 88 molecularly analysed EC plantlets and an additional set of leaves of thirteen extra EC plantlets with dot-like structures on the abaxial leaf surface (S1B Fig), but no endophytic DNA was detected in any of these samples. From the second year onwards, we additionally tested whole leaves of the SC plants and repeated this analysis twice on new leaves of the same SC plants and showed that the majority of the DNA extractions contained DNA of *Burkholderia* (Table 1). The two plantlets that tested negative lacked nodules and we were able to confirm the absence of *Burkholderia* DNA (Table 1). This was also confirmed in the third year, thus establishing their *Burkholderia*-free status.

**Table 1:** Results of the molecular screening of the *in vitro* cultivated EC and SC plantlets. Summary of the DNA extractions from whole leaves of the embryo-cultured (EC) and shoot-cultured (SC) plantlets. After the extractions, the presence of *Burkholderia* DNA was analysed with specific 16S rDNA, *recA*, and *gyrB* primers.

	1^st^ year		2^nd^ year		3^rd^ year	
Presence of bacterial DNA	Positive	Negative	Positive	Negative	Positive	Negative
Embryo-cultured (EC)	0	88	0	88	0	53
Shoot-cultured (SC)	NA	NA	29	2	30	2

To examine possible false negatives, the quality of the DNA extractions and PCR runs were investigated. Each DNA sample was tested for the presence of plant DNA with the *trnL-F* primer. All the analysed DNA samples tested positive on the presence of plant DNA, indicating a successful DNA extraction. In each PCR run, the positive control tested positive for the presence of *Burkholderia* DNA, indicating a successful PCR run.

### Phenotypic analyses

**Fig 3:**
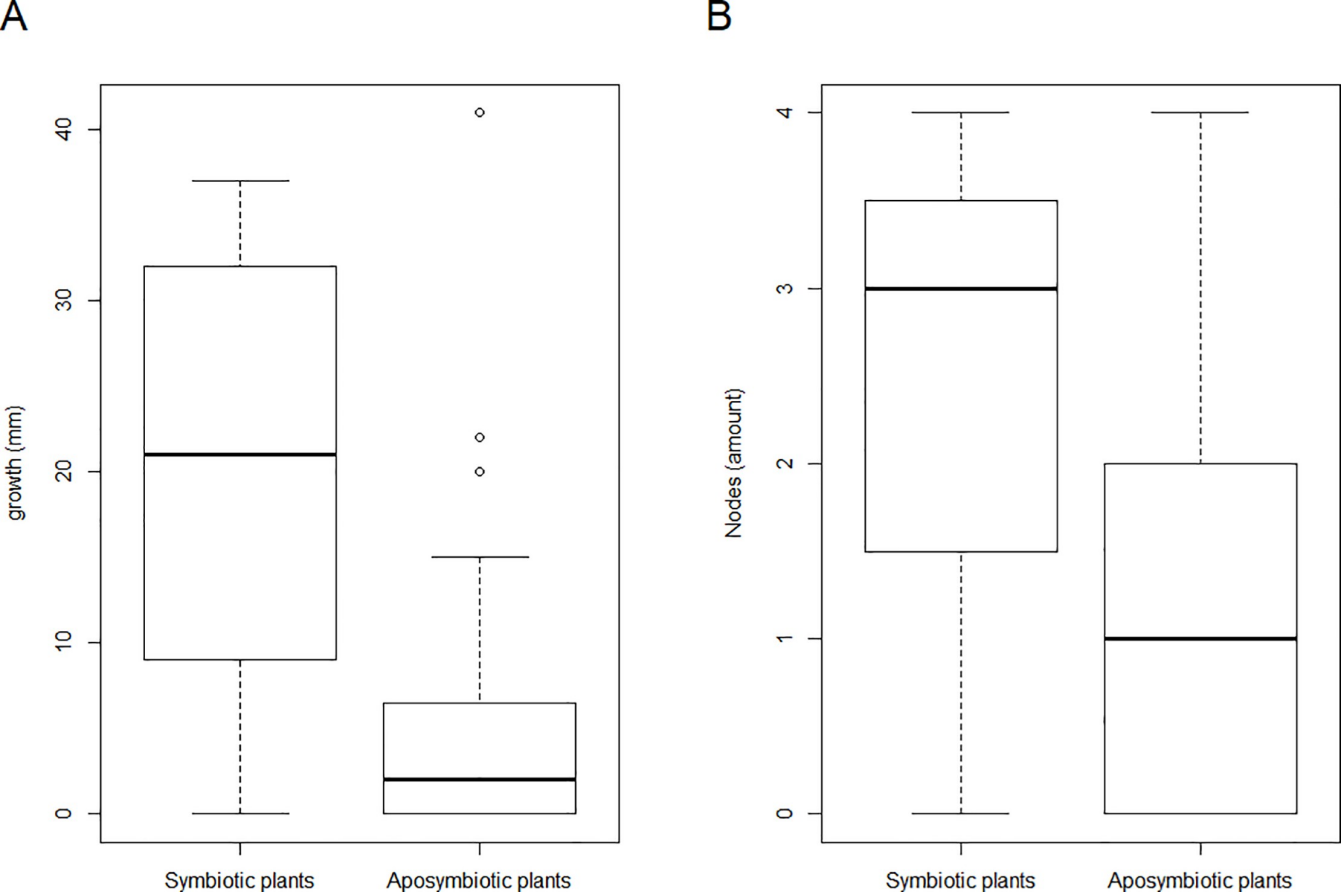
Results of the four-month growth monitoring of aposymbiotic (38 EC+ 1 SC) and symbiotic (15 SC) *P*. *umbellata* plant cuttings. (A) Shoot length monitoring between the first node and the shoot apical meristem (i.e., growth), p = 3.688 x 10^−4^. (B) Development of new nodes in four months, p = 4.715 x 10^−3^.

After two years, we selected 40 EC plantlets (36 from adventive embryony and 4 from germinated embryos) and 24 SC plantlets for phenotypic analyses. Before the experiment, DNA was extracted from the selected plants to test for the presence of endophytic DNA. In 23 of the SC plants endophytic DNA was detected, while no endophytic DNA was detected in all EC plantlets and one SC plantlet. Subsequently, we transferred the shoot (shoot apical meristem and one node) of each plantlet (SC and EC) to new medium (Fig 1I). For four months, we monitored the growth and measured the shoot length starting from the first node above the plant nutrient medium and counted the number of developed nodes every two weeks (S1 Table). During the four-month-period, two of the EC and eight of the SC plantlets were excluded from the analysis due to fungal infection or decay. To analyse the differences in length, and the number of newly-developed nodes, between the aposymbiotic (38 EC + 1 SC) and symbiotic (15 SC) plantlets, a non-parametric Wilcoxon rank test was performed (Fig 3). The aposymbiotic plantlets grew on average 5.4 mm in length, while the symbiotic plantlets grew 20.6 mm in length (S2 Table). Correspondingly, the aposymbiotic plantlets developed on average 1.1 new nodes, while the symbiotic plantlets developed on average 2.4 new nodes (S2 Table). When the individuals without growth or individuals without new nodes were removed, the same conclusions were obtained (S2 Table).

**Fig 4:**
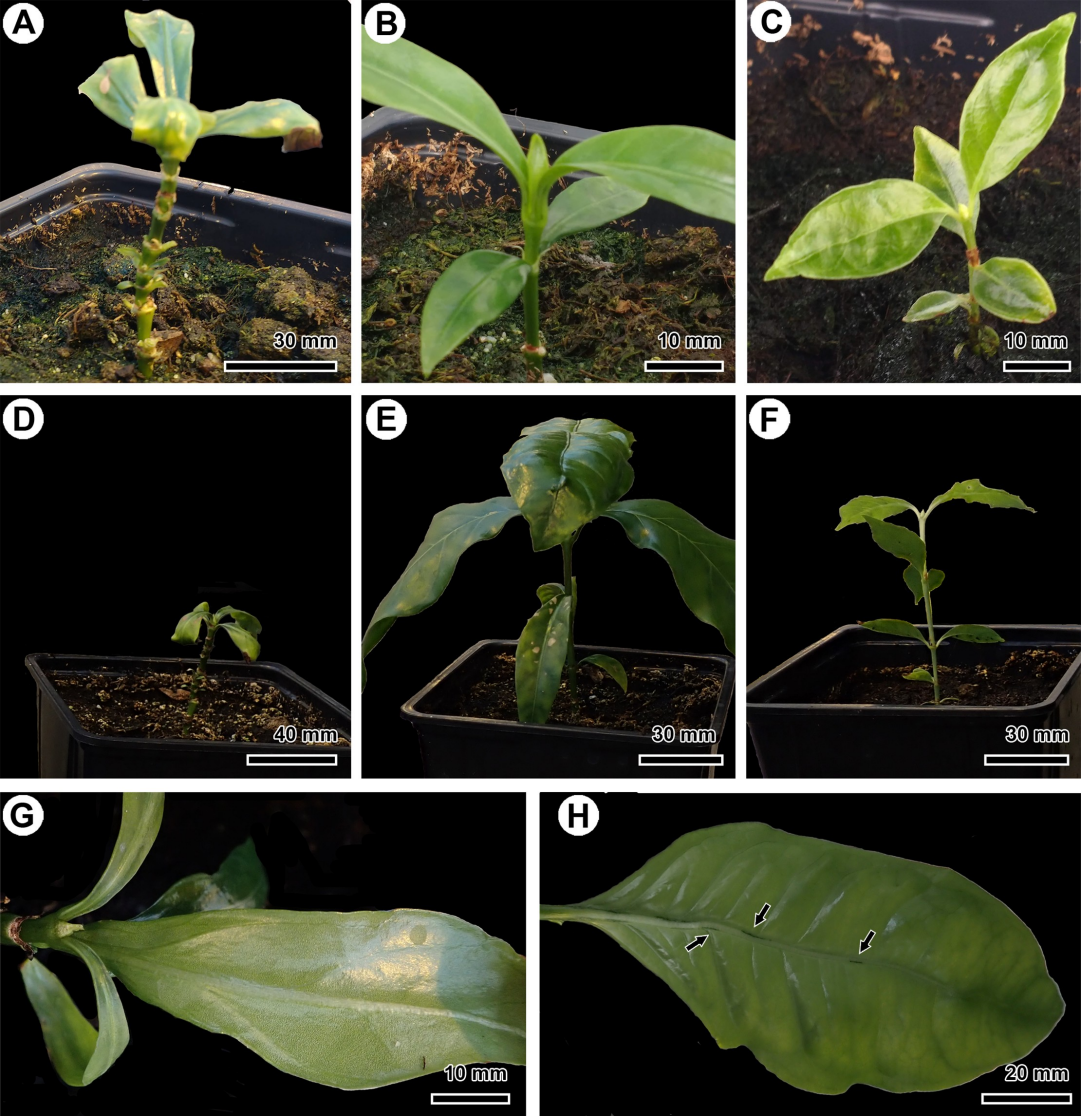
Morphological observations of *in vitro* cultivated *Burkholderia-*free EC plantlets, nodulating SC of *P*. *umbellata* and natural nodule-free *P*. *nervosa* after transfer to soil in greenhouse conditions. (A) EC *P*. *umbellata*, one month after transfer. (B) SC *P*. *umbellata*, one month after transfer. (C) *P*. *nervosa*, one month after transfer. (D) EC *P*. *umbellata*, three months after transfer to the greenhouse. (E) SC *P*. *umbellata*, three months after transfer. (F) *P*. *nervosa*, three months after transfer. (G) Newly developed leaf on an EC plantlet of *P*. *umbellata* without any nodules. (H) Newly developed leaf on the SC plantlet showing nodules under *ex vitro* conditions (arrows).

To further characterise the *Burkholderia*-free phenotype, we transferred the *in vitro* grown plantlets to soil in the greenhouse. This way, we investigated whether these plants survived without their endophyte in an *ex vitro* environment. Eleven *Burkholderia-*free EC plantlets, as well as two nodulating SC plantlets and three *P*. *nervosa*—that is the natural *Burkholderia-*free *Psychotria* species—were transferred to soil (Fig 4A–4C). During the acclimatisation, one of the SC plantlets died due to a fungal infection. After three months, the EC plants did not grow or develop new leaves, in contrast to the SC plant and *P*. *nervosa* that developed two or three new nodes (Fig 4D–4F). After five months, eight EC plantlets died without developing new leaves. Two EC plantlets of *P*. *umbellata* developed one extra node at an axillary shoot and the stipules of these plantlets turned brown after seven months. The new leaves of the EC plantlets were nodule-free (Fig 4G). The SC plantlet of *P*. *umbellata* grew and developed five new nodes, produced new nodulating leaves, and the internodes elongated (Fig 4H). The three EC plantlets of *P*. *nervosa* made on average three to four new nodes and the internodes elongated as well. Furthermore, the stipules of the non-growing EC plants turned brown. This was in contrast to the green stipules covering the apical bud of the nodulating SC plantlets and the apical buds of natural *Burkholderia*-free *P*. *nervosa*.

## Discussion

The use of embryo culture enabled us to develop *Psychotria umbellata* plantlets that are truly *Burkholderia*-free, as confirmed with microscopic and molecular analyses. We showed that these aposymbiotic plantlets survived in a sterile *in vitro* environment, but noticed that their growth and development was significantly slower than for the nodulating plantlets.

Previous research has attempted to develop *Burkholderia*-free plants, too. In Primulaceae, antibiotics and heat shock treatment were applied [20,48], whereas in Rubiaceae, nodule-free plants were observed after seed germination [20,30] or after heat shock treatment [20,31,32]. However, nodulating leaves reappeared after the heat shock treatments, indicating that the endophytes were still present in the axillary buds [31]. In the present study, we used an *in vitro* cultivation technique in order to create *Burkholderia-*free plants and we took several measures to ensure that the endophytes were not latently present. First, we cultivated the EC plants for a long period of time (three years), during which they were continuously monitored. Second, we show that the absence of nodules on the EC plants was not caused by the developmental stage, small leaf size or the *in vitro* environment. Previous studies showed that the first nodules appear on the first leaves of a *P*. *punctata* seedling [20,49], confirming that the developmental stage of the EC plantlets does not explain the missing nodules. We also observed nodules on small leaves of woody adult plants, confirming that the absence of nodules is not caused by the presence of a small leaf size (Fig 2). Furthermore, we detected nodules on the SC plantlets, confirming that the absence of the nodules is not triggered by the *in vitro* environment. The absence of nodules in the leaf lamina of the EC plantlets was also confirmed via light microscopy (Fig 2F). Third, while previous studies only used morphological observations to confirm the absence of the endophyte [20,30,31], we additionally corroborated the *Burkholderia-*free status of the EC plantlets via molecular analysis. Each analysed leaf tested negative for the specific *Burkholderia* primers. To investigate the possibility of false negatives, we repeated this analysis yearly on whole leaves and also tested the vegetative buds for the presence of bacterial DNA. The use of molecular techniques, combined with the long-term monitoring and microscopic observations, provided us with the necessary confidence to confirm that the EC plantlets are truly *Burkholderia*-free and that this condition is permanent. The molecular analysis also confirmed that *in vitro* shoot culture produced two *Burkholderia-*free plantlets. In contrast to the previously used techniques [20,30,31] and the *in vitro* shoot cultivation, all surviving plants obtained through embryo culture are *Burkholderia-*free.

The endophyte is vertically transmitted to the next generation via the seeds, which was confirmed in previous studies in *P*. *punctata* [19–21,25,28]. Despite the presence of the endophytes close to the embryo, embryo culture prevented the transmission to the offspring, which can be explained by several reasons. First, the mucus in the nodulating species of Rubiaceae is assumed to be important for the endophyte as a source of nutrients and a method of transport [20,49,50]. In the seeds of *P*. *punctata*, the cavity between embryo and endosperm is filled with this mucus, which most probably enables the endophytes to survive until the infection of the apical bud [25]. The isolation and subsequent transfer of the embryo to a nutrient-rich medium can influence the presence and/or production of the mucus, which could impact the survival and transport of the endophytes. Second, adventive embryogenesis can prevent infection of the apical buds. The low concentration of endophytes in the seeds [25,30] and the development of new embryos by adventive embryogenesis increases the chance of developing *Burkholderia*-free plants. In *Coffea arabica* (Rubiaceae), it occurs uncommonly *in vitro*, but it is mostly induced during plant cultivation with plant hormones (2,4-dichlorophenoxyacetic acid or naphthalene acetic acid) [51,52]. We observed adventive embryogenesis in 10–30% of the developing embryos after EC in the absence of plant hormones. Third, the nodules of *P*. *umbellata* differ from *P*. *punctata* in size, form (round vs elongated) and position (scattered vs along the midvein) on the leaf lamina [20,43,53]. This variation strongly suggests a different mode of leaf infection by the *Burkholderia* symbiont in *P*. *umbellata*. Besides these variations in nodule morphology and location, the symbiotic cycle of *Candidatus* Burkholderia umbellata can also diverge from the one of *Candidatus* Burkholderia kirkii in its vertical transmission to the next generation, facilitating the development of *Burkholderia-*free seedlings via embryo culture.

Apart from developing *Burkholderia-*free plants of *P*. *umbellata*, we maintained and grew them *in vitro*. In order to evaluate the *Burkholderia-*free phenotype, we cultivated the nodulating plants (SC plants) in the same *in vitro* environment. Our results showed a reduced growth rate when the endophyte is absent. This reduction in growth rate in a sterile controlled environment indicates that leaf nodulation is beneficial for the host plant *P*. *umbellata*. Dwarfed plants without nodules have been observed in Rubiaceae and Primulaceae when they were exposed to hot and dry conditions, or—in small numbers—when they are cultivated from seed [20,30–32,48]. However, in contrast to the crippled phenotype [20,30,31], our plantlets did not produce distorted leaves nor callus at apical meristems (Fig 1D–1F). These features might have been caused by the heat/drought treatments used to produce bacteria-free plants in the previous studies [20,30,31]. Drought is known to cause a reduced growth in *Coffea arabica*, for example [54,55]. To avoid these problems, we opted to use embryo culture and a controlled set-up. *In vitro* cultivation has an effect on the photosynthesis due to the high sugar concentrations and the suboptimal light conditions [47,56]. To take these effects into account, we used a controlled set-up by cultivating natural *Burkholderia*-free *P*. *nervosa* plants via embryo culture and shoot culture of *P*. *umbellata*. The observations of the cultivated *P*. *nervosa* plants using embryo culture suggests that the isolation of the embryo had no effect on the development of the plantlets (Fig 1G and 1H). The cultivation of nodulating SC plants enabled us to compare them with the endophyte-free EC plants in the same sterile *in vitro* environment. To remove possible effects due to the use of a different cultivation technique, shoots of both SC and EC plants were cultivated and monitored. The observation of a reduced growth rate of the shoots of the aposymbiotic plants (EC+1SC) could indicate that the growth rate of the aposymbiotic plantlets is reduced due to the absence of endophytes. Differences in growth rate as a result of genetic variability are negligible since all plantlets are derived from one single specimen. The SC plants are genetic clones of the mother plant and most of the EC plants are derived from adventive embryogenesis. Although the EC plants are the F1 generation of the mother plant and *P*. *umbellata* is a heterostylous species (suggesting out-crossing), self-pollination cannot be excluded under greenhouse conditions.

To characterise the *Burkholderia-*free phenotype under more natural conditions, we transferred some plantlets to greenhouse conditions. Each EC plantlet had a low survival rate and most of the plantlets were not able to produce new leaves or grow normally. This seems to contrast with the nodulating SC plantlet and the natural *Burkholderia-*free *P*. *nervosa* plants that grew, developed new nodes and expanded their leaves. Unfortunately, because we were only able to grow one nodulating SC plantlet, we cannot confirm whether the low survival rate of the aposymbiotic plants is due to the absence of the endophyte or an effect of the acclimatisation to a new environment. Plants that are transferred from *in vitro* to greenhouse conditions need to acclimatise to drier and less nutrient-rich soil by protecting their water content via the stomatal closure and by activating photosynthesis [47,56,57]. These adaptations make the transfer the most critical step for the survival of *in vitro* cultivated plants and a high percentage of plants may get lost [57–61]. Most of the *Burkholderia-*free plantlets did not survive under greenhouse conditions and often died after the degeneration of the shoot apical meristem, or due to low resistance against aphids and fungal infections. Only two plantlets produced an extra pair of nodule-free leaves after four months (Fig 4G). After seven months, the meristem of the axillary shoots degenerated as well. Despite the low number of SC plantlets and EC plantlets of *P*. *nervosa*, our findings demonstrate that nodulating and control plantlets can be transferred from *in vitro* to greenhouse conditions and survive, while the aposymbiotic plantlets cannot (Fig 4). These observations might indicate that the presence of the endophyte is an advantage to adapt to a new environment.

The growth difference between the *Burkholderia*-free and nodulating plantlets in sterile *in vitro* environment indicates that the endophyte might influence plant growth and survival. In nature, many bacterial endophytes that influence the fitness and survival of their hosts have been identified [1,4,7]. They can enhance the growth of the plants via the production of phytohormones, provision of nutrients or reinforce the host’s resilience to biotic or abiotic stress [8–10]. Some of these endophytes can influence the host’s fitness via several pathways. *Burkholderia phytofirmans*, for instance, can increase the host’s biomass by the production of phytohormones or by reducing the abiotic stress (degradation of complex organic compounds, heavy metal efflux mechanisms, *etc*.) [62–65]. During genomic analyses of the *Burkholderia* endophytes of several *Psychotria*, *Pavetta* and *Ardisia* species, known metabolic pathways for growth hormones were not discovered [28,34,66,67]. Instead, it was found that *Candidatus* Burkholderia kirkii produces kirkamide, a secondary compound that might protect the host against insect herbivores [33]. However, this secondary metabolite was not detected in all investigated leaf nodulating plant species; it is for example, not found in the studied specimens of *P*. *umbellata* and *Pavetta schumanniana* [66]. In addition to kirkamide, other glucosides, such as streptol glucoside, were identified in *P*. *punctata* [28,34,66,68]. Streptol glucoside has been shown to inhibit germination of lettuce seeds, which could give the host an allelopathic advantage [66,68]. Although we did not investigate functional pathways within our study to support one of these hypotheses, the growth difference in a controlled nutrient-rich environment (and in absence of any herbivores) suggests that benefits of the bacterial leaf nodulation are not solely ameliorating the host’s defence. For *P*. *umbellata*, the benefits are not yet completely understood and can maybe even differ from those identified in *P*. *punctata*.

In summary, embryo culture had a high success rate to produce *Burkholderia*-free plants of *Psychotria umbellata*. This was corroborated in a three-year survey using observations, and microscopical and molecular techniques. The *in vitro* and greenhouse experiments showed that these plantlets had a lower growth rate compared to the nodulating phenotype, which suggest that the benefits of this intimate interaction are more complex and not yet completely understood. The development of aposymbiotic plants is an important first step to further disentangle the effects of this unique endosymbiosis and embryo culture could facilitate further experimental research due to its high success rate in producing *Burkholderia*-free plants.

## Supporting information

S1 Table Dataset used for statistical analysis of the four-month monitoring of *Burkholderia*-free EC and nodulating SC *P. umbellata* plant cuttings in an *in vitro* environment. Molecular analysis before the start of the experiment confirmed the absence (0) or the presence (1) of *Burkholderia* DNA. After four months, the length difference between the first node and the shoot apical meristem (i.e., growth), and the number of new nodes developed were calculated.
(DOCX)

S2 Table Results of the four-month monitoring of aposymbiotic (38 EC + 1 SC) and symbiotic (15 SC) *P. umbellata* plant cuttings. For both the EC and the SC plantlets, the average length difference between the first node and the shoot apical meristem (i.e., growth), and the average of newly-developed nodes are given. A non-parametric Wilcoxon rank test was used to compare these averages between aposymbiotic (38 EC + 1 SC) and symbiotic (15 SC) plantlets. In addition to the full dataset, the non-parametric Wilcoxon rank test was performed on two subsets. In subset 1, individuals without growth were removed (removal of 12 EC and 1 nodulating SC plantlets), while in subset 2 the individuals without extra nodes were removed (removal of 17 EC and 3 nodulating SC plantlets).
(DOCX)

S1 Fig **Macroscopic and microscopic observation of protruding darker structures on leaves of *Burkholderia*-free EC plantlets (A-C) and nodulating adult plants (D-F) of *P*. *umbellata*.** (A) Nodule-free leaf. (B) Nodule-free leaf showing dark structures at the abaxial lamina surface close to the midvein (arrows). (C) Microscopic detail of a stained transverse vibratome section through one of these structures, confirming that these protruding structures (arrow) are not bacterial nodules. (D) Macroscopic detail of one of the most prominent protruding dark structures (arrow) on the abaxial lamina surface of an adult nodulating leaf. (E) Microscopic detail of a transverse unstained vibratome section through this dark structure showing protrusion of leaf tissue caused by periderm activity (arrow) and the presence of phenolic compounds is suggested by red-brown colouration. (F) UV-autofluorescence of suberin allows distinction of peridermal phellem cells. p, phellem.
(TIF)
