# Supplementary material for: Is the bacterial leaf nodule symbiosis obligate for Psychotria umbellata? The development of a Burkholderia-free host plant
Source: PLoS One. 2019 Jul 16;14(7):e0219863. doi: 10.1371/journal.pone.0219863 (PMC6634412; doi:10.1371/journal.pone.0219863)
Supplement: S1 Table — Molecular analysis before the start of the experiment confirmed the absence (0) or the presence (1) of Burkholderia DNA. After four months, the length difference between the first node and the shoot apical meristem (i.e., growth), and the number of new nodes developed were calculated. (DOCX) [file pone.0219863.s001.docx]

**S1 Table. Dataset used for statistical analysis of the four-month monitoring of *Burkholderia*-free EC and nodulating SC *P. umbellata* plant cuttings in an *in vitro* environment.** Molecular analysis before the start of the experiment confirmed the absence (0) or the presence (1) of *Burkholderia* DNA. After four months, the length difference between the first node and the shoot apical meristem (i.e., growth), and the number of new nodes developed were calculated.

| **ID** | **Cultivation technique** | **Presence endophyte** | **Growth (mm)** | **Development of new nodes** |
| --- | --- | --- | --- | --- |
| 1 | SC | 1 | 0 | 0 |
| 2 | SC | 1 | 3 | 0 |
| 3 | SC | 1 | 5 | 0 |
| 4 | SC | 1 | 30 | 3 |
| 5 | SC | 1 | 15 | 2 |
| 6 | SC | 1 | 37 | 4 |
| 7 | SC | 1 | NA | NA |
| 8 | SC | 1 | NA | NA |
| 9 | SC | 1 | 32 | 3 |
| 10 | SC | 1 | NA | NA |
| 11 | SC | 1 | 7 | 3 |
| 12 | SC | 1 | NA | NA |
| 13 | SC | 1 | 19 | 3 |
| 14 | SC | 0 | 1 | 1 |
| 15 | SC | 1 | 33 | 3 |
| 16 | SC | 1 | NA | NA |
| 17 | SC | 1 | NA | NA |
| 18 | SC | 1 | 21 | 4 |
| 19 | SC | 1 | 28 | 1 |
| 20 | SC | 1 | NA | NA |
| 21 | SC | 1 | 11 | 4 |
| 22 | SC | 1 | 36 | 2 |
| 23 | SC | 1 | 32 | 4 |
| 24 | SC | 1 | NA | NA |
| 1 | EC | 0 | 1 | 1 |
| 2 | EC | 0 | 6 | 2 |
| 3 | EC | 0 | 1 | 0 |
| 4 | EC | 0 | 0 | 0 |
| 5 | EC | 0 | 9 | 2 |
| 6 | EC | 0 | 4 | 1 |
| 7 | EC | 0 | 14 | 3 |
| 8 | EC | 0 | NA | NA |
| 9 | EC | 0 | 0 | 0 |
| 10 | EC | 0 | 20 | 2 |
| 11 | EC | 0 | 22 | 4 |
| 12 | EC | 0 | 4 | 1 |
| 13 | EC | 0 | 1 | 0 |
| 14 | EC | 0 | 0 | 2 |
| 15 | EC | 0 | 0 | 0 |
| 16 | EC | 0 | 0 | 0 |
| 17 | EC | 0 | 0 | 0 |
| 18 | EC | 0 | 4 | 2 |
| 19 | EC | 0 | 6 | 1 |
| 20 | EC | 0 | 0 | 0 |
| 21 | EC | 0 | 2 | 0 |
| 22 | EC | 0 | 15 | 3 |
| 23 | EC | 0 | 0 | 0 |
| 24 | EC | 0 | 0 | 0 |
| 25 | EC | 0 | 3 | 0 |
| 26 | EC | 0 | 3 | 1 |
| 27 | EC | 0 | 7 | 2 |
| 28 | EC | 0 | 41 | 4 |
| 29 | EC | 0 | 7 | 2 |
| 30 | EC | 0 | 0 | 0 |
| 31 | EC | 0 | 2 | 1 |
| 32 | EC | 0 | NA | NA |
| 33 | EC | 0 | 2 | 1 |
| 34 | EC | 0 | 4 | 0 |
| 35 | EC | 0 | 0 | 0 |
| 36 | EC | 0 | 0 | 0 |
| 37 | EC | 0 | 2 | 0 |
| 38 | EC | 0 | 15 | 3 |
| 39 | EC | 0 | 2 | 1 |
| 40 | EC | 0 | 13 | 1 |
