# Supplementary material for: Is the bacterial leaf nodule symbiosis obligate for Psychotria umbellata? The development of a Burkholderia-free host plant
Source: PLoS One. 2019 Jul 16;14(7):e0219863. doi: 10.1371/journal.pone.0219863 (PMC6634412; doi:10.1371/journal.pone.0219863)
Supplement: S2 Table — For both the EC and the SC plantlets, the average length difference between the first node and the shoot apical meristem (i.e., growth), and the average of newly-developed nodes are given. A non-parametric Wilcoxon rank test was used to compare these averages between aposymbiotic (38 EC + 1 SC) and symbiotic (15 SC) plantlets. In addition to the full dataset, the non-parametric Wilcoxon rank test was performed on two subsets. In subset 1, individuals without growth were removed (removal of 12 EC and 1 nodulating SC plantlets), while in subset 2 the individuals without extra nodes were removed (removal of 17 EC and 3 nodulating SC plantlets). (DOCX) [file pone.0219863.s002.docx]

**S2 Table: Results of the four-month monitoring of aposymbiotic (38 EC + 1 SC) and symbiotic (15 SC) *P. umbellata* plant cuttings.** For both the EC and the SC plantlets, the average length difference between the first node and the shoot apical meristem (i.e., growth), and the average of newly-developed nodes are given. A non-parametric Wilcoxon rank test was used to compare these averages between aposymbiotic (38 EC + 1 SC) and symbiotic (15 SC) plantlets. In addition to the full dataset, the non-parametric Wilcoxon rank test was performed on two subsets. In subset 1, individuals without growth were removed (removal of 12 EC and 1 nodulating SC plantlets), while in subset 2 the individuals without extra nodes were removed (removal of 17 EC and 3 nodulating SC plantlets).

|  | **Full dataset** | | | **Subset 1** | | | **Subset 2** | | |
| --- | --- | --- | --- | --- | --- | --- | --- | --- | --- |
|  | **Aposymbiotic** | **Symbiotic** | **p-values** | **Aposymbiotic** | **Symbiotic** | **p-values** | **Aposymbiotic** | **Symbiotic** | **p-values** |
| Growth (mm) | 5.4 | 20.6 | 1.159 x 10^-4^ | 7.8 | 22.1 | 4.421 x 10^-4^ | 8.318 | 25.1 | 2.327 x 10^-4^ |
| Development of new nodes | 1.1 | 2.4 | 3.721 x 10^-3^ | 1.3 | 2.6 | 8.532 x 10^-3^ | 1.9 | 3 | 4.508 x 10^-3^ |
