# Supplementary figures and images for: Is the bacterial leaf nodule symbiosis obligate for Psychotria umbellata? The development of a Burkholderia-free host plant
Source: PLoS One. 2019 Jul 16;14(7):e0219863. doi: 10.1371/journal.pone.0219863 (PMC6634412; doi:10.1371/journal.pone.0219863)

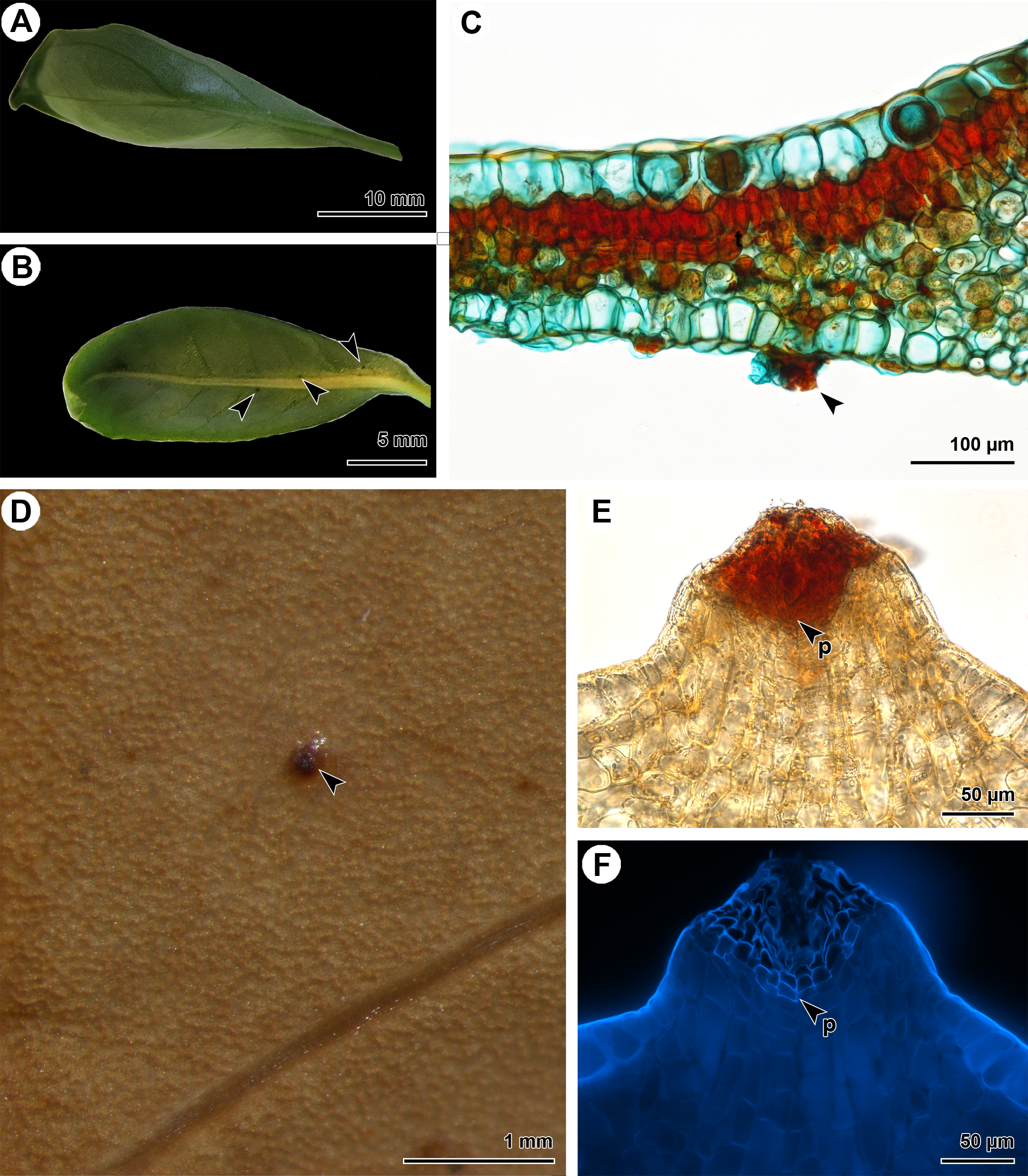

Supplement: S1 Fig — Macroscopic and microscopic observation of protruding darker structures on leaves of Burkholderia-free EC plantlets (A-C) and nodulating adult plants (D-F) of P. umbellata. (A) Nodule-free leaf. (B) Nodule-free leaf showing dark structures at the abaxial lamina surface close to the midvein (arrows). (C) Microscopic detail of a stained transverse vibratome section through one of these structures, confirming that these protruding structures (arrow) are not bacterial nodules. (D) Macroscopic detail of one of the most prominent protruding dark structures (arrow) on the abaxial lamina surface of an adult nodulating leaf. (E) Microscopic detail of a transverse unstained vibratome section through this dark structure showing protrusion of leaf tissue caused by periderm activity (arrow) and the presence of phenolic compounds is suggested by red-brown colouration. (F) UV-autofluorescence of suberin allows distinction of peridermal phellem cells. p, phellem. (TIF) [file pone.0219863.s003.tif]
